# Vitamin C transporter SVCT1 serves a physiological role as a urate importer: functional analyses and in vivo investigations

**DOI:** 10.1007/s00424-023-02792-1

**Published:** 2023-02-07

**Authors:** Yu Toyoda, Hiroshi Miyata, Naohiro Uchida, Keito Morimoto, Ryuichiro Shigesawa, Hidetoshi Kassai, Kazuki Nakao, Naoko H. Tomioka, Hirotaka Matsuo, Kimiyoshi Ichida, Makoto Hosoyamada, Atsu Aiba, Hiroshi Suzuki, Tappei Takada

**Affiliations:** 1 Department of Pharmacy, The University of Tokyo Hospital, 7-3-1 Hongo, Bunkyo-ku, Tokyo, 113-8655 Japan; 2 Laboratory of Animal Resources, Center for Disease Biology and Integrative Medicine, Graduate School of Medicine, The University of Tokyo, 7-3-1 Hongo, Bunkyo-ku, Tokyo, 113-0033 Japan; 3 Department of Human Physiology and Pathology, Faculty of Pharma-Sciences, Teikyo University, 2-11-1 Kaga, Itabashi-ku, Tokyo, 173-8605 Japan; 4 Department of Integrative Physiology and Bio-Nano Medicine, National Defense Medical College, 3-2 Namiki, Tokorozawa, Saitama 359-8513 Japan; 5 Department of Pathophysiology, Tokyo University of Pharmacy and Life Sciences, 1432-1 Horinouchi, Hachioji, Tokyo, 192-0392 Japan

**Keywords:** Anti-oxidant, Ascorbic acid, Protein evolution, *Svct1* KO, Uricosuric agent

## Abstract

Uric acid, the end product of purine metabolism in humans, is crucial because of its anti-oxidant activity and a causal relationship with hyperuricemia and gout. Several physiologically important urate transporters regulate this water-soluble metabolite in the human body; however, the existence of latent transporters has been suggested in the literature. We focused on the *Escherichia coli* urate transporter YgfU, a nucleobase-ascorbate transporter (NAT) family member, to address this issue. Only SLC23A proteins are members of the NAT family in humans. Based on the amino acid sequence similarity to YgfU, we hypothesized that SLC23A1, also known as sodium-dependent vitamin C transporter 1 (SVCT1), might be a urate transporter. First, we identified human SVCT1 and mouse Svct1 as sodium-dependent low-affinity/high-capacity urate transporters using mammalian cell-based transport assays. Next, using the CRISPR-Cas9 system followed by the crossing of mice, we generated *Svct1* knockout mice lacking both *urate transporter 1* and *uricase*. In the hyperuricemic mice model, serum urate levels were lower than controls, suggesting that *Svct1* disruption could reduce serum urate. Given that Svct1 physiologically functions as a renal vitamin C re-absorber, it could also be involved in urate re-uptake from urine, though additional studies are required to obtain deeper insights into the underlying mechanisms. Our findings regarding the dual-substrate specificity of SVCT1 expand the understanding of urate handling systems and functional evolutionary changes in NAT family proteins.

**Supplementary Information:**

The online version contains supplementary material available at 10.1007/s00424-023-02792-1.

## Introduction

The global increase in the incidence of gout [13, 25], the most prevalent form of inflammatory arthritis caused by hyperuricemia which is the sustained elevation of serum urate (the predominant form of uric acid under physiological conditions), has highlighted the significance of urate maintenance at optimal levels for a healthy life. The urate-degrading enzyme *uricase* (*UOX*) is genetically absent in humans [57], and uric acid is the final product of purine metabolism. Since urate cannot passively traverse cellular membranes, active transport plays a pivotal role in regulating urate handling in the human body, suggesting the physiological significance of urate transporter proteins. Indeed, other groups and we have identified physiologically important urate transporters of which functional changes influence serum urate levels, such as renal urate re-absorbers—urate transporter 1 (URAT1, also known as SLC22A12) [14] and glucose transporter 9 (GLUT9/SLC2A9) [8, 32, 52], and an intestinal and renal urate exporter—ATP-binding cassette transporter G2 (ABCG2) [21, 33, 56]. However, the previously described urate transporters do not thoroughly explain human urate handling systems. This suggests the presence of latent urate transporters in the human body. As a reference point for investigating such mechanisms, we focused on prokaryotic (non-mammalian) proteins exhibiting urate transport activity.

YgfU (NCBI accession; NP_417364.2), a member of the nucleobase-ascorbate transporter (NAT) family, was previously identified as a low-affinity and high-capacity urate transporter in *Escherichia coli* [37]. Given the functional similarities between homologous genes, we hypothesized that mammalian proteins homologous to YgfU might be involved in urate transport. Consistent with the fact that only SLC23A proteins are classified as members of the NAT family in humans [15], an in silico homology search conducted using the BLAST-P program (accessed in July 2013) revealed that SLC23A1 and SLC23A2 are the closest to YgfU in amino acid sequence, leading us to focus on them as a source for latent urate transporter(s).

SLC23A1 and SLC23A2 are sodium-dependent vitamin C transporters (SVCT1 and SVCT2, respectively) [50] involved in the cellular uptake of vitamin C (L-ascorbic acid) in various tissues/organs [6, 38, 42]. A previous study using *Svct1* knockout (KO) mice suggested that SVCT1, which is expressed in the brush border of proximal tubules in the kidney [7, 26], is involved in the re-absorption of vitamin C from urine into the blood [11]. Despite its widespread distribution [54], SVCT2 is hardly expressed in the kidney [9, 26]. *Svct2* KO mice exhibit low vitamin C levels in tissues and die soon after birth [39], demonstrating the physiological relevance of SVCT2. Despite the abundance of research on SVCTs, we could not find any information on whether SVCTs can transport urate or participate in urate handling in the human body. To address this concern, we focused on SVCT1/SLC23A1 and investigated its function as a urate transporter from molecular and physiological perspectives.

This study aimed to investigate the urate transport ability of human SVCT1 and mouse Svct1 using cell-based transport assays. Hyperuricemic *Svct1* KO mice deficient in both *Urat1* and *Uox* were generated using the CRISPR-Cas9 system. In addition, we performed functional assays to explore the effects of serum urate (SU)-affecting drugs and their active metabolites (a total of 28 compounds) on SVCT1-mediated urate and vitamin C transport. Our findings provide insights into the dual-substrate specificity of SVCT1 and its physiological implications.

## Materials and methods

### Materials

The critical materials and resources used in this study are summarized in Supplementary Table S1. pCAG-EGxxFP (Addgene plasmid # 50716), pCAG-EGxxFP-Cetn1 (Addgene plasmid # 50717), and pX330-Cetn1/1 (Addgene plasmid # 50718) were gifts from Masahito Ikawa [30]. Fuji Yakuhin (Saitama, Japan) kindly provided dotinurad under a material transfer agreement. The remaining chemicals were of analytical grade and commercially available. Detailed information on SU-affecting drugs and their active metabolites used in this study is summarized in Supplementary Table S2.

### Plasmid construction

The full-length human SVCT1/SLC23A1 wild-type (WT) (NCBI accession; NM_005847.5) open reading frame (ORF) was inserted into the pEGFP-N1 vector (Clontech Laboratories, Palo Alto, CA, USA) using *EcoR*I and *Sal* I sites. A site-directed mutagenesis technique removed the original termination codon and achieved in-frame fusion with a downstream EGFP protein (i.e., SVCT1-EGFP expression). An expression vector with mouse Svct1/Slc23a1 WT (NCBI accession; NM_011397.4) was constructed similarly. Exon 6 was excluded from the Svct1-EGFP expression vector using the site-directed mutagenesis technique to express the Svct1 KO mutant.

The full-length human ORFs, URAT1/SLC22A12 WT (NCBI accession; NM_144585.3) in pEGFP-C1 [48] and human GLUT9a/SLC2A9a WT (NCBI accession, NM_020041.2) in pEGFP-N1 [44], were derived from our previous studies. All plasmid constructs were confirmed by sequencing using BigDye^®^ Terminator v3.1 (Applied Biosystems, Foster City, CA, USA) on an Applied Biosystems^®^ 3130 Genetic Analyzer (Applied Biosystems), according to the manufacturer’s guidelines. All plasmids used in the experiments were originated from the same batch.

### Cell culture

Human embryonic kidney-derived HEK293 cells were maintained in Dulbecco’s Modified Eagle’s Medium (Nacalai Tesque, Kyoto, Japan) supplemented with 10% fetal bovine serum (Biowest, Nuaillé, France), 1% penicillin-streptomycin (Nacalai Tesque), 2 mM L-glutamine (Nacalai Tesque), and 1 × non-essential amino acid (Life Technologies, Tokyo, Japan) at 37°C in a humidified atmosphere of 5% (v/v) CO_2_ in air. In addition, HEK-derived 293A cells and Madin-Darby canine kidney II (MDCKII) cells were also maintained similarly.

For in vitro assays, each vector plasmid was transfected into HEK293 cells on 12-well cell culture plates 24 h after seeding (0.92 × 10^5^ cells/cm^2^) using a forward transfection approach with polyethyleneimine “MAX” (PEI-MAX) (Polysciences, Warrington, PA, USA) as described previously [34]. For microscopic observation, HEK293 cells were seeded on collagen-coated glass-bottom dishes (Matsunami Glass, Tokyo, Japan). After 24 h of incubation, the medium was replaced with a fresh medium. Inhibitory tests with SU-affecting drugs and their active metabolites were performed on 293A cells.

For z-stack microscopic observation of polarized cells, MDCKII cells were seeded on collagen-coated glass-bottom dishes at a density of 1.31 × 10^5^ cells/cm^2^ and transiently transfected with the respective plasmid vectors using PEI-MAX (2.0 μg plasmid/10 μL of PEI-MAX/dish) as described previously [35].

### Animals

All animal experiments were conducted according to methods approved by the Institutional Animal Care and Use Committee of The University of Tokyo. All animals received humane care following the criteria outlined in the Guide for the Care and Use of Laboratory Animals prepared by the National Academy of Sciences and published by the National Institutes of Health. The animals were maintained on a standard FR-1 diet (Funabashi Farm, Chiba, Japan) with ad libitum water, under 12-h light/dark cycles, as described previously [40].

*Uox* KO mice on a C57BL/6J genetic background were obtained from the Jackson Laboratory (Bar Harbor, ME, USA) (Stock No: 002223, B6;129S7-Uox^tm1Bay^/J) [58], *Urat1*-*Uox* double knockout (DKO) mice were obtained from our earlier investigations [17, 18, 55]. In addition, drinking water was supplemented with 180 mg/L allopurinol (FUJIFILM Wako Pure Chemical, Osaka, Japan) and 4 g/L Uralyt-U^®^ (a standardized mixture of potassium citrate and sodium citrate,Nippon Chemiphar, Tokyo, Japan) to attenuate hyperuricemia phenotypes that cause severe renal impairment in mice with *Uox*^−/−^ genetic background, as described previously [47].

*Svct1*-*Urat1*-*Uox* triple knockout (TKO) mice were generated using the CRISPR-Cas9 system [36], according to our previous studies [35, 47]. Briefly, single guide RNAs (sgRNAs) for the *Svct1* gene disruption were designed using CRISPRdirect (https://crispr.dbcls.jp/, accessed in December 2015), each sgRNA was evaluated using an EGxxFP system [31]. For this purpose, HEK293 cells were transiently co-transfected with the evaluated sgRNA/pX330, genomic fragments containing sgRNA target sequence (approximately 320 bp)/pCAG-EGxxFP, and pCAG-mRFP. Based on the in vitro analyses (detailed in the “Results” section), we determined the target sequence for genome editing: 5′-ccagggtgcaatcatggtgtcca-3′ (detailed in the “Results” section). Next, the synthesized sgRNA (FASMAC, Kanagawa, Japan) and in vitro transcribed Cas9 mRNA were microinjected into mouse zygotes fertilized in vitro (WT ovum × *Uox* KO sperm), the microinjected embryos were transferred into recipient mice. We focused on a 19-bp deletion between intron 5 and exon 6 in the *Svct1* gene found in *Uox*^+/−^ F0 mice. After confirming the germ-line transmission of the deletion allele, the founder mouse was mated with WT mice (C57BL/6J, Japan SLC, Shizuoka, Japan) and *Uox* KO mice to generate *Svct1* KO mice and *Svct1*-*Uox* DKO mice by the further crossing of mice, respectively. The latter mice were further mated with *Urat1*-*Uox* DKO mice to generate *Svct1*-*Urat1*-*Uox* TKO mice.

Genotyping of the 19-bp deletion was conducted during *Svct1* KO allele isolation, using a TA cloning-based direct sequencing, as described previously [35]. After the generation of each mouse line, *Svct1* genotypes were determined using specific PCR primer sets. Similar PCR-based methods were used to genotype *Uox* and *Urat1* KO alleles. Information on each PCR primer is summarized in Supplementary Table S3.

### Specimen collection

Spot urine samples were collected on a plastic wrap sheet and transferred to fresh tubes. Under anesthesia with isoflurane (FUJIFILM Wako Pure Chemical), blood was drawn from the jugular veins, and plasma or serum was obtained as described previously [35, 45]. All liquid specimens were stored at −80°C until further processing. Immediately following euthanasia, tissues were excised, weighed, and rapidly frozen in liquid nitrogen, and stored until subsequent analysis.

### Preparation of protein lysates and immunoblotting

Whole-cell lysate (WCL) samples were prepared as described previously [35]. At 48 h post-plasmid transfection, HEK293 cells were washed twice with ice-cold potassium-free phosphate-buffered saline [PBS (−)] and lysed in ice-cold RIPA lysis buffer [50 mM Tris-HCl, pH 7.4, 150 mM NaCl, 0.1% sodium dodecyl sulfate (SDS), 0.5% sodium deoxycholate, 1% NP-40, 1 mM phenylmethylsulfonyl fluoride, and a Protease Inhibitor Cocktail for General Use (Nacalai Tesque)]. The solution was centrifuged at 15,000 × *g* at 4°C for 10 min, and the supernatant was collected in a separate tube. The protein concentration of the WCL was quantified using a Pierce™ BCA Protein Assay Kit (Thermo Fisher Scientific, Carlsbad, CA, USA) with bovine serum albumin (BSA) as a standard, according to the manufacturer’s protocol.

Protein extracts from mouse tissues were prepared in ice-cold RIPA lysis buffer using the methods in our previous study [45]. Immediately following euthanasia, tissues were excised, weighed, and then homogenized using a Physcotron homogenizer (Microtec, Chiba, Japan). Crude lysates were incubated at 4°C for 1 h with gentle rotation, and following the centrifugation at 15,000 × *g* at 4°C for 10 min, the resulting supernatant was collected in a fresh tube. The protein concentration was determined the Pierce™ BCA Protein Assay Kit.

Immunoblot analyses were performed with minor modifications from our previous study [35]. The loading samples were separated by SDS polyacrylamide gel electrophoresis and electroblotted on a PVDF membrane (Immobilon-P, Millipore, Bedford, MA, USA) at 15 V for 60 min. The membrane was blocked by incubation in Tris-buffered saline containing 0.05% Tween 20 and 5% skim milk (TBST-5%SM). Blots were probed with the specific antibodies diluted in TBST-5%SM (Supplementary Table S1), and an HRP-dependent luminescence was developed using ECL™ Prime Western Blotting Detection Reagent (GE Healthcare, Buckinghamshire, UK). Immunocomplexes were detected using a multi-imaging Analyzer Fusion Solo 4™ system (Vilber Lourmat, Eberhardzell, Germany).

### Confocal microscopic observation

Confocal laser-scanning microscopy was conducted as described previously [35]. Post 48 h of plasmid transfection, cells were fixed with ice-cold methanol. After washing with PBS (–), the cells were treated with TO-PRO-3 iodide (Molecular Probes, Eugene, OR, USA) to visualize nuclei. After a subsequent wash with PBS (–), the cells were mounted on Fluorescence Mounting Medium (Agilent, Santa Clara, CA, USA). Fluorescence was detected using the FV10i Confocal Laser Scanning Microscope (Olympus, Tokyo, Japan) to analyze the localization of EGFP-fused transporter proteins.

### Cell-based transport assay

We conducted cell-based urate uptake assays using human SVCT1- or mouse Svct1-expressing HEK293 cells 48 h after plasmid transfection with minor modifications from our previous studies [35, 47] to evaluate their latent urate transport activities. Krebs–Ringer buffer (133 mM NaCl, 4.93 mM KCl, 1.23 mM MgSO_4_, 0.85 mM CaCl_2_, 20 mM CAPS, 5 mM D-glucose, 5 mM L-glutamine, at pH 7.4 unless otherwise indicated, when using a Na^+^-free condition, NaCl was replaced with equimolar choline chloride) was employed as a transport buffer. After pre-incubation in the transport butter at 37°C for 15 min, the cells were incubated for 10 min or indicated periods in pre-warmed fresh transport buffer containing [8-^14^C]-uric acid (53 mCi/mmol; American Radiolabeled Chemicals, St. Louis, MO, USA) at 10 μM or specified concentrations. SVCT1-inhibiting effects of the target compounds, including vitamin C, were examined using Krebs–Ringer buffer either without (i.e., with only vehicle control) or with the individual compounds at the indicated concentrations. The cells were washed twice with ice-cold transport buffer and lysed with 500 μL of 0.2 M NaOH on ice. The lysates were neutralized with 100 μL of 1 M HCl. A liquid scintillator (Tri-Carb 3110TR; PerkinElmer) was used to measure the radioactivity of the lysates. Protein concentrations in the lysates were assessed using the Pierce™ BCA Protein Assay Kit.

The urate transport activity was calculated as incorporated clearance (μL/mg protein/min) = (incorporated level of urate [DPM/mg protein/min] / urate level in the incubation mixture [DPM/μL]). Subtracting the urate transport activity of mock cells from that of human SVCT1- and mouse Svct1-expressing cells determined their respective urate transport activities. The vitamin C transport activity was calculated similarly based on data obtained using [1-^14^C]-vitamin C (L-ascorbic acid) (7.3 mCi/mmol; PerkinElmer, Waltham, MA, USA) at 20 μM as a substrate.

Similar uptake assays were performed using URAT1-expressing HEK293 cells with a Cl^−^-free transport buffer [Cl^−^-free Hanks’ balanced salt solution: 125 mM Na-gluconate, 4.8 mM K-gluconate, 1.2 mM KH_2_PO_4_, 1.2 mM MgSO_4_, 1.3 mM Ca-gluconate, 25 mM HEPES, 5.6 mM D-glucose, at pH 7.4] according to our previous studies [34, 48]. In cell-based assays for GLUT9a, a high-potassium transport buffer (145.4 mM KCl, 0.8 mM MgSO_4_, 1.8 mM CaCl_2_, 25 mM HEPES, 25 mM Tris, 5 mM D-glucose, and pH 7.4) was employed.

### Calculation of the kinetic parameters and half-maximal inhibitory concentration values

The Michaelis-Menten constant (*K*_m_) and maximal velocity (*V*_max_) of the SVCT1- or Svct1-mediated urate transport were determined by fitting the Michaelis–Menten model to experimental urate transport rates and concentrations using nonlinear regression curve fitting in GraphPad Prism 8 (GraphPad Software, San Diego, CA, USA), as described previously [49].

The urate and vitamin C transport activities were evaluated in the presence of several target compound concentrations to calculate their half-maximal inhibitory concentration (IC_50_) values against urate and vitamin C transport by SVCT1. The SVCT1-mediated transport activities were expressed as a percentage of the control (100%). Using the least-squares method with Excel 2019 (Microsoft, Redmond, WA, USA), fitting curves were obtained based on the calculated values according to the following formula as described previously [49]:

$$\mathrm{Predicted\, value\, }\left[\mathrm{\%}\right]=100-\left({~}^{{\mathrm{E}}_{\mathrm{max}}\times {\mathrm{C}}^{\mathrm{n}}}\!\left/ \!{~}_{{\mathrm{EC}}_{50}^{\mathrm{n}}+{\mathrm{C}}^{\mathrm{n}}}\right.\right)$$

where E_max_ is the maximum effect, EC_50_ is the half-maximal effective concentration, C is the concentration of the test compound, and n is the sigmoid-fit factor. Finally, based on these results, the IC_50_ was calculated.

### Vesicle transport assay using ABCG2-expressing plasma membrane vesicles

An in vitro vesicle transport assay [46] was conducted using ABCG2-expressing plasma membrane vesicles for the functional characterization of ABCG2. As described previously [34], the ABCG2-expressing plasma membrane vesicles and control vesicles were prepared from 293A cells infected with EGFP-ABCG2-expressing adenovirus and control (EGFP) adenovirus, respectively. Then, the transport assay was conducted using a rapid filtration technique [34]. Vesicles (0.5 mg/mL) were incubated at 37°C for 10 min in the presence of [8-^14^C]-urate (10 μM) or [1-^14^C]-vitamin C (500 μM), in a reaction mixture (10 mM Tris/HCl, 250 mM sucrose, 10 mM MgCl_2_, 10 mM creatine phosphate, 1 mg/mL creatine phosphokinase, pH 7.4, and 50 mM ATP or AMP as an ATP substitute). After washing by ice-cold stop buffer five times, radioactivity incorporated into the vesicles was detected using the liquid scintillator (Tri-Carb 3110TR). The transport activity was calculated as an incorporated clearance (mL/mg protein/min): (incorporated level of tested compound [DPM/mg protein/min] / tested compound level in the reaction mixture [DPM/mL]).

### Experimental measurement of vitamin C by LC-PDA analysis

As described previously [23, 35], vitamin C concentrations in WT or *Svct1* KO mice plasma were determined using liquid chromatography-photodiode array (LC-PDA) analysis with an ACQUITY UPLC^®^ PDA Detector (Waters, Milford, MA, USA) coupled with an ACQUITY UPLC System (Waters). After deproteinization with equal volumes of 10% (w/v) metaphosphate solution containing 1 mM EDTA, each sample was centrifuged at 20,000 × *g* at 4°C for 10 min,the obtained supernatant was diluted with equal volumes of 25 mM phosphate buffer (pH 2.1) containing 60 μM acyclovir (FUJIFILM Wako Pure Chemical) as an internal control and then separated on a CAPCELL PAK ADME S3 column (maintained at 50°C; 3 μm, 2.1 × 100 mm; Osaka Soda, Osaka, Japan) with a gradient mobile phase (0–4 min: 2% B; 4–5 min: 2–98% B; 5–9 min: 98% B; 9–10 min: 98–2% B; 10–12 min: 2% B) of 25 mM phosphate buffer (pH 2.1) (A) and methanol (B) at a flow rate of 300 μL/min. Vitamin C and acyclovir were measured at 243 nm in the PDA spectrum. Calibration curves for the analyte were generated using a series of murine plasma spiked with L-ascorbic acid (the reduced form of vitamin C) standard solutions. Peaks were analyzed using MassLynx NT software v4.1 (Waters).

### Experimental measurement of urate and creatinine by LC-MS/MS analysis

Serum and urinary urate and creatinine concentrations were determined using an LC-mass spectrometry (MS)/MS system comprising an ACQUITY UPLC^®^ instrument coupled to a Xevo TQ-S triple-quadrupole MS/MS system (Waters), as described in previous investigations [19, 41], with some modifications. After adding acyclovir as an internal standard, specimens were purified by protein precipitation with four volumes of methanol, specimens were diluted in some cases. Each preprocessed sample was separated on an ACQUITY UPLC BEH C18 Column (maintained at 50°C; 1.7 μm, 2.1 × 150 mm; Waters) using gradient mobile phases: [0–3.5 min: 0% B; 3.5–5 min: 0–5% B; 5–5.5 min: 5–95% B; 5.5–10.5 min: 95% B; 10.5–11 min: 95–0% B; 11–15 min: 0% B] of 0.1% formic acid in water (A) and 0.1% formic acid in acetonitrile (B) at a flow rate of 300 μL/min for urate and [0–5 min: 10% D; 5–6 min: 10–90% D; 6–11 min: 90% D; 11–12 min: 90–10% D; 12–15 min: 10% D) of 5 mM ammonium acetate in water (C) and 0.1% formic acid in methanol (D) at a flow rate of 200 μL/min for creatinine. Ionization was performed using a heated electrospray ionization probe and analytes under optimized conditions for each target compound (Supplementary Table S4), and target compounds were monitored in multiple reactions monitoring mode. Calibration curves for the analyte were generated from a series of standard solutions of uric acid and creatinine. Peak analyses and quantification were conducted using MassLynx NT software version 4.1.

### Calculation of fractional excretion of uric acid

Fractional excretion of uric acid (urate clearance/creatinine clearance ratio, FE_UA_) was calculated using the serum and urine concentrations of urate and creatinine mentioned above, using the following formula: FE_UA_ = (urine urate concentration [μM]/serum urate concentration [μM]) / (urine creatinine concentration [μM]/serum creatinine concentration [μM]) × 100 [%].

### Statistics

All statistical analyses were performed using Excel 2019 with Statcel4 add-in software (OMS publishing, Saitama, Japan). The number of biological replicates (*n*) and the different statistical tests conducted for each experiment are detailed in figure legends. The similarity of variance across multiple groups was compared using Bartlett’s test. When passing the test for homogeneity of variance, a parametric Tukey–Kramer multiple-comparison test for all pairwise comparisons or a parametric Williams’ multiple-comparison test for trend analysis was used. In the case of a single pair of quantitative data, after comparing the variances of a set of data using an *F*-test, an unpaired Student’s *t*-test was performed. *P* values less than 0.05 or 0.01 were considered statistically significant.

All experiments were monitored in a non-blinded fashion. Samples that experienced technical failure during processing were excluded from analyses. Each experiment used the minimum number of mice or samples required to obtain informative results and sufficient data for subsequent studies.

## Results

### Identification of human SVCT1 as a urate transporter

**Fig. 1:**
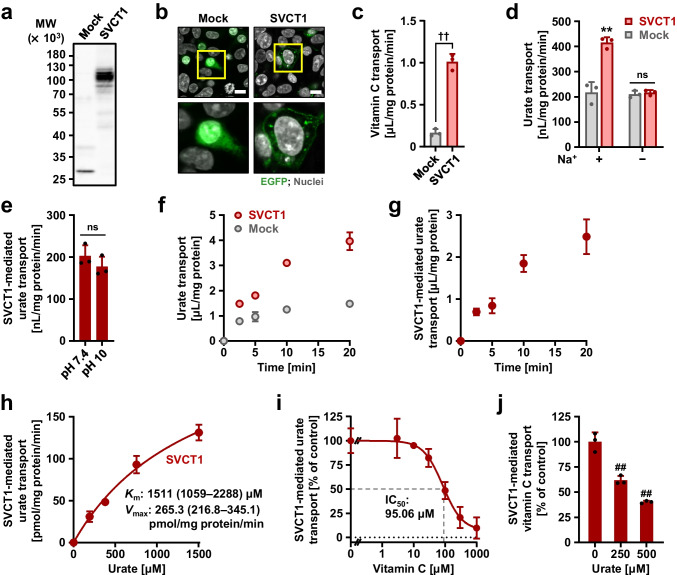
Identification and characterization of human SVCT1 as a urate transporter. All uptake assays were conducted using transiently SVCT1-expressing HEK293 cells 48 h after plasmid transfection in Krebs–Ringer buffer. Experimental conditions: pH 7.4 unless otherwise indicated, or pH 10 (*h*); [1-^14^C]-vitamin C in the transport buffer was 20 μM (*c*, *j*); incubation time and [8-^14^C]-urate in the transport buffer were 5 min and 10 μM, respectively, unless otherwise indicated. **a–c** Functional expression of SVCT1 in HEK293 cells 48 h after the transfection. (*a*) Immunoblot detection of SVCT1 protein in whole-cell lysates. (*b*) Intracellular localization of SVCT1 detected by confocal microscopy. Magnified images of representative cells denoted by yellow squares are demonstrated. Scale bars: 10 μm. (*c*) [1-^14^C]-Vitamin C transport activities into the cells. **d–i** SVCT1 as a urate transporter. (*d*) Sodium-dependency in SVCT1-mediated urate transport. (*e*) Insignificant effect of an alkaline pH condition (pH 10) on the urate transport activity of SVCT1. (*f*) Time-dependent [8-^14^C]-urate incorporation into SVCT1-expressing or mock (control) cells. (*g*) Time profile for SVCT1-mediated [8-^14^C]-urate uptake into HEK293 cells, which was calculated by subtracting the urate transport activity of mock cells from that of SVCT1-expressing cells. (*h*) Concentration dependence in SVCT1-mediated [8-^14^C]-urate transport. Regarding the estimated Michaelis-Menten constant (*K*_m_) and maximal velocity (*V*_max_), the 95% confidence interval values were provided in parentheses. (*I*) Concentration-dependent inhibition of SVCT1-mediated urate transport by vitamin C. IC_50_, the half-maximal inhibitory concentration. **j** Inhibitory effect of urate on SVCT1-mediated vitamin C transport at physiological concentrations. Values are shown as % of vehicle control (*i*, *j*). Data are expressed as the mean ± SD; where vertical bars are not displayed, the SD was contained within the limits of the symbol; *n* = 3. ^††^*P* < 0.01 (two-sided *t*-test; *c*, *e*); ns, not significantly different between groups; ^**^P < 0.01 *vs.* the other groups (Tukey–Kramer multiple-comparison test; *d*); ^##^*P* < 0.01 *vs.* vehicle control (Williams’ multiple-comparison test; *j*)

We evaluated the urate transport ability of SVCT1 using mammalian HEK293 cells transiently expressing SVCT1. Immunoblotting and confocal microscopy confirmed the expression (Fig. 1a) and plasma membrane localization (Fig. 1b) of EGFP-tagged SVCT1 in the cells. When incubated in Krebs–Ringer buffer mimicking blood ionic content, the SVCT1-expressing cells incorporated [1-^14^C]-vitamin C more efficiently than mock cells (Fig. 1c), demonstrating the functional expression of SVCT1. In a similar experimental condition, [8-^14^C]-urate transport into the SVCT1-expressing cells was also significantly greater than mock cells; however, Na^+^ exclusion from the Krebs–Ringer buffer blocked the SVCT1-mediated urate transport (Fig. 1d). These results indicated that SVCT1 is a sodium-dependent urate transporter. A high pH condition (the Krebs–Ringer buffer at pH 10) had minimal effect on the SVCT1-mediated urate transport activity (Fig. 1e).

Based on the results of time-course experiments (Fig. 1f, g), we investigated urate uptake at 5 min in subsequent analyses to determine the initial rates of SVCT1-mediated transport. Because uric acid is sparingly soluble at neutral pH, kinetic analyses were conducted at high pH to achieve high urate concentrations [up to 1.5 mM (approximately 25 mg/dL)] in the buffer during uptake experiments (Fig. 1h); the calculated *K*_m_ and *V*_max_ were 1511 μM and 265.3 pmol/mg protein/min, respectively, for SVCT1-mediated urate transport. Given this affinity indicator value, in terms of substrate self-inhibition, physiological levels of circulating urate [120–420 μM (approximately 2–7 mg/dL) in a healthy person] could have no significant effect on SVCT1-mediated urate transport. However, vitamin C inhibited urate transport activity in a concentration-dependent manner, with an IC_50_ value of 95.06 μM (Fig. 1i), suggesting that physiological levels of circulating vitamin C (50–90 μM in a healthy person with ≥100 mg of daily vitamin C intake [27]) would affect SVCT1-mediated urate transport. Moreover, 500 μM urate decreased SVCT1-mediated vitamin C transport to approximately 40% of the vehicle control (Fig. 1j). These findings indicate a potential interaction between urate and vitamin C mediated by SVCT1 in humans.

**Fig. 2:**
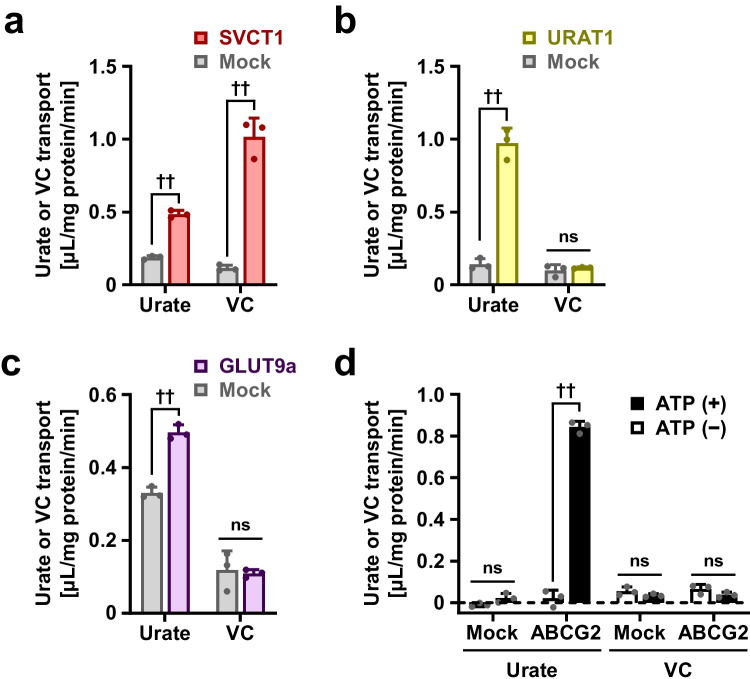
Investigation of vitamin C transport activities in physiologically important urate transporters. Regarding SVCT1 (**a**), URAT1 (**b**), GLUT9a (**c**), and ABCG2 (**d**), [8-^14^C]-urate or [1-^14^C]-vitamin C transport activities were investigated. SVCT1 exhibited a dual substrate specificity, whereas URTA1, GLUT9a, and ABCG2 lacked vitamin C transport activities. Cell-based uptake assays were conducted in Krebs–Ringer buffer (*a*), Cl^−^-free Hanks’ balanced salt solution (*b*), or high-potassium transport buffer (*c*); incubation time for uptake was 5 min. In vitro transport assay was carried out using ABCG2-expressing plasma membrane vesicles; incubation time for uptake was 10 min (*d*). Data are expressed as the mean ± SD; *n* = 3. ^††^*P* < 0.01; ns, not significantly different between groups (two-sided *t*-test)

Considering the dual functionality of SVCT1 as a vitamin C and urate transporter (Fig. 2a), we investigated vitamin C transport activity in three well-characterized physiologically important urate transporters: URAT1 (Fig. 2b), GLUT9 (Fig. 2c), and ABCG2 (Fig. 2d). Even under appropriate experimental conditions in which urate transport activities were detected, neither URAT1, GLUT9, nor ABCG2 could transport vitamin C.

### Identification of mouse Svct1 as a urate transporter

**Fig. 3:**
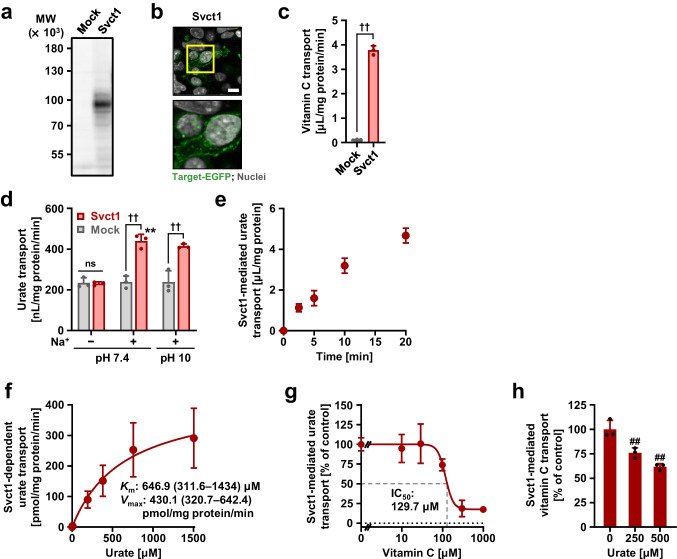
Identification and characterization of mouse Svct1 as a urate transporter. All uptake assays were conducted using transiently Svct1-expressing HEK293 cells 48 h after plasmid transfection in Krebs–Ringer buffer. Experimental conditions: pH 7.4 unless otherwise indicated, or pH 10 (*f*); [1-^14^C]-vitamin C in the transport buffer was 20 μM (*c*, *h*); incubation time and [8-^14^C]-urate in the transport buffer were 5 min and 10 μM, respectively, unless otherwise indicated. **a–c** Functional expression of Svct1 in HEK293 cells 48 h after the transfection. (*a*) Immunoblot detection of Svct1 protein in whole-cell lysates. (*b*) Intracellular localization of Svct1 detected by confocal microscopy. Magnified images of representative cells denoted by yellow squares are demonstrated. Scale bar: 10 μm. (*c*) [1-^14^C]-Vitamin C transport activities into the cells. **d–g** Svct1 as a urate transporter. (*d*) Sodium-dependency in Svct1-mediated urate transport, which was insignificantly affected by an alkaline pH condition (pH 10). (*e*) Time profile for Svct1-mediated [8-^14^C]-urate uptake into HEK293 cells. (*f*) Concentration dependence in Svct1-mediated [8-^14^C]-urate transport. Regarding the estimated Michaelis-Menten constant (*K*_m_) and maximal velocity (*V*_max_), the 95% confidence interval values were provided in parentheses. (*g*) Concentration-dependent inhibition of Svct1-mediated urate transport by vitamin C. IC_50_, the half-maximal inhibitory concentration. **h** Mild inhibitory effect of urate on Svct1-mediated vitamin C transport at physiological concentrations. Values are shown as % of vehicle control (*g*, *h*). Data are expressed as the mean ± SD; where vertical bars are not displayed, the SD was contained within the limits of the symbol; *n* = 6 (*f*), 3 (*the others*). ^††^*P* < 0.01; ns, not significantly different between groups (two-sided *t*-test; *c*, *d*); ^**^P < 0.01 *vs.* the other groups for pH 7.4 (Tukey–Kramer multiple-comparison test; *d*); ^##^*P* < 0.01 *vs.* vehicle control (Williams’ multiple-comparison test; *h*)

We investigated the urate transport activity of mouse Svct1 in vitro (Fig. 3) prior to the generation of *Svct1* KO mice. Functional expression of Svct1 in HEK293 cells was confirmed by immunoblotting (Fig. 3a), confocal microscopy (Fig. 3b), and vitamin C transport assay (Fig. 3c). Our findings confirmed that Svct1 is a sodium-dependent urate transporter of which urate transport activity was maintained even at high pH (Fig. 3d). Since the time profile of Svct1-mediated urate transport (Fig. 3e) was quite similar to SVCT1 (Fig. 1g), kinetic analyses for Svct1 (Fig. 3f) were conducted under identical experimental conditions. Svct1-mediated urate transport had a *K*_m_ of 646.9 μM and a *V*_max_ of 430.1 pmol/mg protein/min, suggesting no significant difference in molecular characteristics between human SVCT1 and mouse Svct1. Vitamin C also inhibited Svct1-mediated urate transport (similar to SVCT1) with an IC_50_ value of 129.7 μM (Fig. 3g). In addition, 500 μM urate decreased the Svct1-mediated vitamin C transport to approximately 60% of the vehicle control (Fig. 3h), indicating that Svct1 might be more resistant to the inhibitory effect of urate on vitamin C transport activity than SVCT1.

### Generation and analyses of *Svct1* KO mice with a genetic background of *Uox* and *Urat1* deficiency

**Fig. 4:**
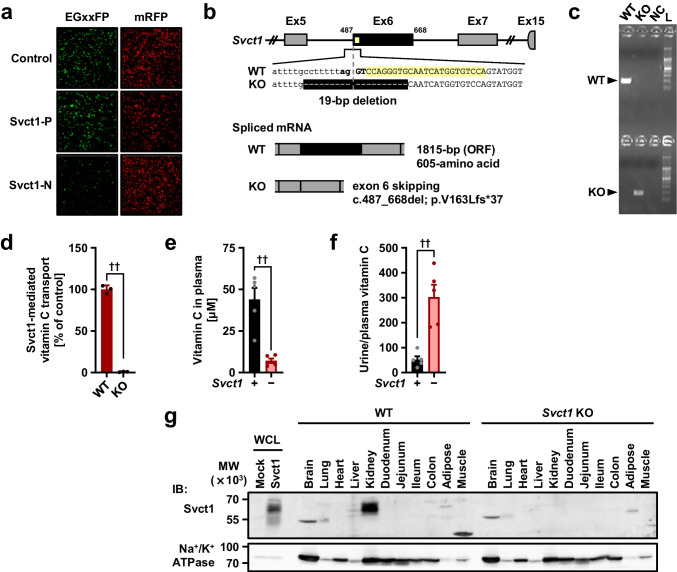
Generation and validation of *Svct1* knockout mice. **a** An EGxxFP-based in vitro evaluation of sgRNAs for *Svct1* knockout. Control, previously validated sgRNA sequence targeting Centrin 1 as a positive control for the EGxxFP system; Svct1-P, sgRNA sequence targeting *Svct1* performed the best in this study (5′-ccagggtgcaatcatggtgtcca-3′); Svct1-N, a representative sgRNA sequence targeting *Svct1* performed poorly in this study (5′-catgaggtcgtggattcagcagg-3′). **b** Schematic illustration of the location of 19-bp deletion in the *Svct1* gene. The 19-bp deletion was found at the boundary of intron 5 and exon 6 (Ex6); this deletion is theoretically expected to result in exon 6 skipping, resulting in the frameshift variant p.V163Lfs*37. WT, wild-type; KO, knockout; ORF, open reading frame. **c** Representative results of PCR-based genotyping for each knockout allele in *Svct1* KO mice. NC, non-template control; L, 100-bp ladder marker. **d** Functional validation of Svct1 KO variant. The KO variant, Svct1 p.V163Lfs*37, transiently expressed in HEK293 cells was ineffective as a vitamin C transporter in Krebs–Ringer buffer (pH 7.4) containing 20 μM [1-^14^C]-vitamin C. Data are expressed as the mean ± SD, *n* = 3. **e**, **f** Vitamin C levels in plasma (*e*) and calculated urine-to-plasma vitamin C concentration ratios (*f*) of *Svct1* single KO mice. Data are expressed as the mean ± SEM, *n* = 5. ^††^*P* < 0.01 (two-sided *t*-test; *d*–*f*). **g** Immunoblot detection of Svct1 in each murine tissue using the anti-Svct1 antibody. Whole cell lysate (WCL) samples prepared from Svct1-expressing and mock HEK293 cells 48 h after transfection were used as positive and negative controls, respectively. A strong signal for Svct1 was detected in the kidney of WT mice but not in *Svct1* KO mice, indicating the successful knockout of *Svct1* in this study; a side-by-side comparison is shown in Supplementary Fig. S1. Na^+^/K^+^ ATPase, a marker for plasma membrane components

Next, we generated *Svct1* KO mice to examine the involvement of Svct1 in urate handling (Fig. 4). Contrary to humans, uric acid is further metabolized by Uox in WT mice; thus, the *Uox* KO genetic background was used in this study to address urate levels in vivo. As described in the “Materials and methods” section, genome editing was conducted using the CRISPR-Cas9 system with a functional gRNA targeting the exon 6 of *Svct1* (Fig. 4a), resulting in a 19-bp deletion including exon/intron boundary sequences located on the front of exon 6 (Fig. 4b, c). Given that this type of mutation leads to single exon skipping [3], the deletion must have resulted in c.487–688del (p.V163Lfs*37). This frameshift mutation disrupted the vitamin C transport activity of Svct1 (Fig. 4d). Moreover, the loss-of-function was confirmed in vivo in terms of vitamin C handling (Fig. 4e, f) and Svct1 protein expression (Fig. 4g). Indeed, *Svct1* KO mice exhibited lower vitamin C levels in plasma (Fig. 4e) and higher values of the urine/plasma vitamin C concentration ratio (Fig. 4f) than WT mice. Immunoblotting successfully detected the renal Svct1 expression, which was absent in *Svct1* KO mice (Fig. 4g, Supplementary Fig. S1). These results were consistent with a previous report showing that Svct1 is involved in the re-uptake of vitamin C from urine into the blood by the kidneys [11]. Using MDCKII cells, a polarized renal cell line, we confirmed the apical localization of both human SVCT1 and mouse Svct1 (Supplementary Fig. S2).

**Fig. 5:**
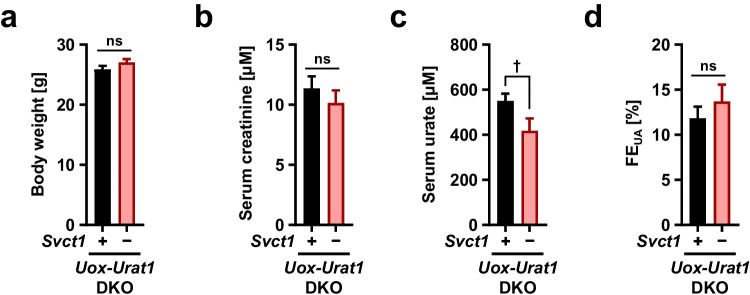
Effects of *Svct1* knockout on urate handling in mice deficient in *Urat1* and *Uox*. Body weight (**a**), serum creatinine (**b**), serum urate (**c**), and fractional renal urate excretion (FE_UA_, urate clearance/creatinine clearance ratio) (**d**) in *Svct1*-*Urat1*-*Uox* triple knockout (TKO) mice and control [*Urat1*-*Uox* double knockout (DKO)] mice. Serum urate levels in TKO mice were significantly lower than DKO mice. Data are expressed as the mean ± SEM, *n* = 14 (DKO) and 10 (TKO). ^†^*P* < 0.05; ns, not significantly different between groups (two-sided *t*-test)

The physiological function of Svct1 as a vitamin C re-absorber expressed on the apical membrane of renal epithelial cells prompted us to hypothesize that Svct1 might also serve as a renal urate re-absorber. Given that in healthy conditions, Urat1 is the most influential apical mechanism in the kidney, we knocked out *Urat1* from *Svct1*-*Uox* DKO mice to sharpen the potency of Svct1, followed by in vivo analyses of the resulting *Svct1*-*Urat1*-*Uox* TKO mice (Fig. 5). The TKO mice exhibited no significant differences in body weight (Fig. 5a), or serum creatinine (a renal function marker) concentrations (Fig. 5b) from control *Urat1*-*Uox* DKO mice, demonstrating that Svct1 deficiency did not induce severe renal dysfunction. Serum urate concentrations in the TKO mice were significantly lower than in the control DKO mice (Fig. 5c), indicating the physiological impact of Svct1 on urate handling. Although FE_UA_ values were not significantly different between the two lines (Fig. 5d), an increasing trend (13.7% in TKO; 11.8% in DKO) was consistent with Svct1 deficiency reducing serum urate.

### Investigation of inhibitory effects of serum urate-affecting drugs on SVCT1-mediated urate and vitamin C transport

**Fig. 6:**
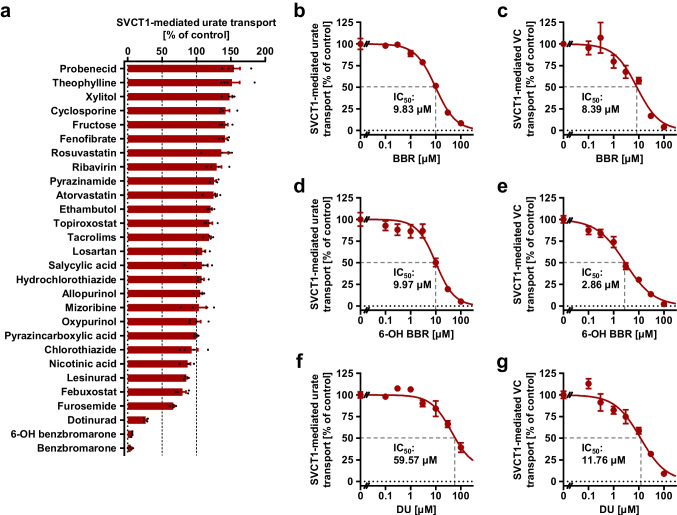
Effects of serum urate-affecting drugs and their active metabolites on the urate and vitamin C transport activities of SVCT1. Forty-eight h after transfection, SVCT1-expressing 293A cells were subjected to a cell-based urate or vitamin C (VC) transport assay. The transport activities were measured for 2.5 min in the presence of each compound at the indicated concentrations. BBR, benzbromarone; 6-OH BBR, 6-hydroxybenzbromarone; DU, dotinurad; IC_50_, the half-maximal inhibitory concentration. **a** The effects of each compound [experimentally maximum concentrations (refer to Supplementary Table S2)] on the urate transport activities of SVCT1. Comparison of the effects on the urate and VC transport activities of SVCT1 was shown in Supplementary Fig. S3. **b–g** Concentration-dependent inhibition of SVCT1-mediated urate (*b*, *d*, *f*) and VC (*c*, *e*, *g*) transport by BBR, 6-OH BBR, and DU. Values are shown as % of vehicle control (0.1% dimethyl sulfoxide); data are expressed as the mean ± SEM; *n* = 4

Some SU-affecting drugs have been previously reported to inhibit physiologically important urate transporters with clinical implications via off-target interactions, including febuxostat–ABCG2 and losartan–organic anion transporter 10 (OAT10) [34, 49]. However, there was no information on their effects on SVCT1. Therefore, we investigated the effects of SU-affecting drugs and their active metabolites (a total of 28 compounds listed in Supplementary Table S2) on SVCT1 function as a urate and vitamin C transporter (Fig. 6, Supplementary Fig. S3).

An in vitro screening was conducted at maximum drug concentrations (3–1000 μM; Supplementary Table S2). Although some tested drugs caused a moderate enhancement of the urate transport activities of SVCT1 (Fig. 6a), inhibitory effects on SVCT1-mediated urate and vitamin C were comparable in most cases (Supplementary Fig. S3). Benzbromarone, 6-hydroxybenzbromarone, and dotinurad reduced urate transport activities of SVCT1 to <50% of the vehicle controls (Fig. 6a). The IC_50_ values were then calculated by measuring urate and vitamin C transport activities at different concentrations of each compound (Fig. 6b–g). The calculated IC_50_ values against urate and vitamin C transport activities of SVCT1 for benzbromarone, 6-hydroxybenzbromarone, and dotinurad were 9.83 μM and 8.39 μM, 9.97 μM and 2.86 μM, and 59.57 μM and 11.76 μM, respectively.

Next, the IC_50_ values of each compound were compared to its estimated maximum unbound concentration in human plasma (f_u_C_max_) (Supplementary Table S5). We calculated the ratio of f_u_C_max_/IC_50_ as an indicator, whose high value (≥1) suggested a possible inhibition of SVCT1 by each compound in clinical settings. The calculated values were much lower than 1 for benzbromarone and dotinurad. Despite the lack of 6-hydroxybenzbromarone pharmacokinetic data in humans, the pharmacokinetic parameters of 6-hydroxybenzbromarone and benzbromarone were similar in a prior investigation on monkeys [43]. Our results suggested that neither benzbromarone, 6-hydroxybenzbromarone, nor dotinurad, in circulation impaired SVCT1 function.

## Discussion

In this study, we performed in vitro cell-based assays (Figs. 1 and 3) followed by in vivo approaches using hyperuricemic mice harboring the genetic background of *Svct1* KO (Figs. 4 and 5) to investigate SVCT1 as a candidate for mammalian urate transporter based on its similarity to YgfU, a prokaryote urate transporter. Given the sodium-dependence of SVCT1-mediated transport and the higher sodium ion concentrations in external fluids relative to the cytosol, SVCT1 must be involved in cellular urate uptake under physiological conditions. Also, we found that urate and vitamin C could compete for SVCT1-mediated transport (Fig. 1i, j). Since both urate and vitamin C serve significant roles as anti-oxidants in the body [1], comprehending their potential interactions in maintaining stable levels may contribute to a more extensive understanding of biological homeostasis regulation.

Our findings suggest that SVCT1 is not specific to vitamin C. These results contradict a widely accepted notion regarding mammalian NATs, which were considered highly specific for vitamin C because SVCTs did not recognize nucleobases as substrates [15, 24, 50]. Traditionally, NAT proteins have been divided into three groups based on their substrate specificities: the first is exclusive to bacteria and specific for uracil,the second is present in bacteria, fungi, and plants and specific for nucleobases and oxidized purines, such as xanthine and/or uric acid (urate); the third is found in vertebrates and specific for vitamin C (L-ascorbate) [15]. Consequently, microbial and plant NATs are nucleobase transporters but not vitamin C transporter, whereas vertebrate NATs have the opposite substrate specificity. All mammalian NATs, except for rat Slc23a4, the first sodium-dependent nucleobase transporter (SNBT1), failed to display substrate selectivity for nucleobases such as uracil and xanthine [50, 54, 59]. However, rSnbt1/Slc23a4 was not involved in vitamin C transport [59], and orthologues of rat *Snbt1*/*Slc23a4* are pseudogenes in almost all primates, including humans [24]. Thus, evidence obtained herein that SVCT1 recognizes both vitamin C and urate (an oxidized purine) as substrates suggests that primate SVCTs could have retained some functional aspects of their ancestral proteins that must have been nucleobase transporters.

YgfU, a homolog of SVCT1 in *Escherichia coli*, functions as a urate transporter [37], indicating that specific ancestral urate transporters acquired vitamin C transport abilities during evolution. Inhibitory effects of vitamin C on UapA (a well-studied fungus NAT)-mediated xanthine transport [24] suggest a potential molecular interaction between nucleobase-specific NAT(s) and vitamin C under very low-affinity recognition. Thus, primate SVCTs might have evolved from ancestral nucleobase transporters, retaining a portion of their original molecular properties as nucleobase transporters while also acquiring vitamin C transport abilities. Considering a hypothesis that this functional acquisition might have resulted from the evolutional optimization of the sub-function of ancestral proteins [24], such a shift in substrate specificity of NATs (nucleobases–vitamin C) may reflect an evolutionary alternation in the significance of these compounds for each species.

The *K*_m_ values of SVCT1 for urate [1511 μM, human SVCT1 (Fig. 1h); 647 μM, mouse Svct1 (Fig. 3f)] were higher than those for vitamin C established in previous studies (65–237 μM, human SVCT1 [38], 29 μM, rat Svct1 [50]. SVCT1, therefore, has a strong affinity for vitamin C than urate. The results of this study imply that SVCT1 could have a greater physiological significance as a vitamin C transporter than as a urate transporter, as genetic disruption of *Svct1* had a greater impact on vitamin C levels in the blood rather than urate (Figs. 4e and 5c). Nevertheless, our current study deepens the understanding of urate handling systems.

The physiological roles of SVCT1 should be discussed, accompanied by some limitations and perspectives of this study. Further studies are required to understand the mechanism by which SVCT1 regulates serum urate levels, a crucial aspect of urate management. We hypothesized that Svct1 might serve as a renal urate re-absorber based on its high expression in the kidney (Fig. 4g) and function as a renal vitamin C re-absorber [11]. Given the effects of genetic dysfunction on FE_UA_ in humans, URAT1 has the greatest contribution on the net amount of renal urate re-uptake from the luminal side of the proximal tubule among renal urate re-absorbers (including unidentified transporters) [12, 20]. Hence, this study employed the genetic background of *Urat1* KO to emphasize the potential function. As expected, serum urate levels in *Svct1*-*Urat1*-*Uox* TKO mice were lower than in control *Urat1*-*Uox* DKO mice (Fig. 5c), while there was no statistical difference in the values of FE_UA_ (an indicator for renal urate clearance) between the mice groups (Fig. 5d). To bridge this gap, we can envision a several possibilities as follows.

Since the effects of *Svct1*-knockout on urate handling was not sufficiently large (but significant in serum urate), sample size used in this study could have not allow us to detect a statistically significant difference in FE_UA_ (calculated by four variables—serum urate concentration and the other three parameters; details are in the “Materials and methods” section). Based on this assumption, our results suggest that SVCT1 could have a role as the third member of renal urate re-absorbers expressed on the apical side of renal proximal tubular cells following URAT1 and OAT10 (a recently identified physiologically important machinery) [4, 16, 49]. Due to their different urate *K*_m_ values—371 μM, URAT1 [14], 558 μM, OAT10 [49], 1511 μM, SVCT1 (Fig. 1h), when the high-affinity/low-capacity transporter (URAT1) is saturated or dysfunctional, the other low-affinity/high-capacity transporters might compensate. As we have previously proposed with regard to OAT10 [49], this notion for the presence of compensation/back up system(s) is consistent with clinical features of renal hypouricemia which is a pathological condition characterized by low serum urate (≤ 120 μM) and increased renal urate elimination (decreased renal urate reabsorption)—in renal hypouricemia type 2 related to the genetic dysfunction of GLUT9 (a basal machinery involved in urate secretion from renal tubular cells into the blood), typical values of FE_UA_ are extremely high (≥ 100%), in renal hypouricemia type 1 related to that of URAT1, such FE_UA_ values are high (tens of %) but relatively lower than those in type 2 [22]. This difference in FE_UA_ would reflect the contribution of apical machineries for urate re-uptake other than URAT, however, all the remained cannot be explained by OAT10 alone, given the effect of its dysfunction on renal urate handling revealed by a clinico-genetic analysis [49]. Accordingly, SVCT1 could be a component of the systems that have not been entirely identified yet.

Moreover, further supportive information is available. Renal proximal tubules are divided into S1–S3 segments that reportedly have various roles (reabsorption, secretion, and post-secretory reabsorption, respectively) in renal urate handling [29]. Recent transcriptome analysis of murine renal tubule segments [9] revealed that all the three urate transporters were highly expressed in the S2 and S3 segments (Supplementary Fig. S4), however, contrary to *Oat10*, *Urat1* and *Svct1* were expressed also in the S1 segment. Although the segmental distribution of each protein in mouse and human kidneys needs to be validated, such expression profiles can strengthen the notion for the backup systems. In addition, previous studies reported that SVCT1-mediated vitamin C transport activity was significantly lower at low (acidic) pH conditions [50, 54], a similar and comparable effect (reduction in relative transport activity) was found in SVCT1-mediated urate transport activity (Supplementary Fig. S5). Contrary to SVCT1, cellular function of OAT10 was significantly higher at such condition [49]. Given these molecular properties in pH sensitivity, physiological contributions of SVCT1 and OAT10 on urate handling might be altered according to pH in primary urine. Thus, addressing what differences in the impact on urate handling exist between SVCT1 and OAT10 will be a future issue.

The latent involvement of non-renal Svct1 may provide an alternate explanation for the TKO mice phenotype. A previous study using *Svct1* KO mice showed that accumulation levels of an orally-administered vitamin C analog (6-bromo-6-deoxy-L-ascorbic acid, an SVCT1 substrate [10] in intestinal mucosa were comparable between WT and *Svct1* KO mice, suggesting that Svct1 could only have a limited effect on the intestinal vitamin C absorption [11]. The contribution of Svct1-mediated intestinal urate absorption to serum urate levels is unlikely in this context, given Svct1 expression was not detected elsewhere other than the kidney by immunoblotting (Fig. 4g).

Additionally, although vitamin C intestinal absorption has been characterized as an SVCT1-mediated mechanism in recent literature [28, 51], there is no evidence regarding the molecular machinery involved in this process in humans. This speculation seemed plausible at the onset of SVCT1 research due to the expression of SVCT1 mRNA in the intestine [54] and the apical localization of transfected SVCT1 protein in polarized Caco-2 cells (a human colon carcinoma-derived cell line) [5]. However, *Svct1* KO mice were still capable of absorbing vitamin C from the diet [11], and a non-functional splice variant of SVCT1 (which cannot been distinguished with a functional variant by northern blotting) was expressed in normal human enterocytes [53], therefore, the role of SVCT1 in mediating the intestinal vitamin C absorption should be carefully interpreted. Our findings on the chemical inhibition of SVCT1-mediated vitamin C transport (Fig. 6) may be relevant pharmacologically. A pharmacokinetic model of gastrointestinal absorption [2] suggests that maximal clinical dosages of benzbromarone (50 mg) and dotinurad (4 mg) can result in maximum concentrations in the intestinal lumen of 379 μM and 35.9 μM, respectively, which are sufficient for substantial inhibition of SVCT1.

In conclusion, we established that SVCT1 functions as a sodium-dependent urate transporter, of which genetic disruption decreased serum urate levels in a hyperuricemic mouse model. Although further studies are needed to unravel the physiological mechanisms underlying the phenotype, our findings contribute to a better understanding of urate handling systems. Furthermore, given the dual-substrate specificity of SVCT1, this NAT family transporter could be a key molecule for elucidating a latent interaction between urate and vitamin C in the human body.

## Supplementary Information

Below is the link to the electronic supplementary material.

Supplementary file1 (DOCX 272 KB)

## Data Availability

Data are available from the corresponding author upon reasonable request. All data relevant to the study are included in the article or uploaded as online Supplementary Information.

## Abbreviations

ABCG2: ATP-binding cassette transporter G2
BSA: Bovine serum albumin
DMSO: Dimethyl sulfoxide
DKO: Double knockout
FE_UA_: Fractional excretion of uric acid
GLUT: Glucose transporter
IC_50_: Half-maximal inhibitory concentration
HEK: Human embryonic kidney
MDCKII: Madin-Darby canine kidney II
*V*_max_: Maximal velocity
f_u_C_max_: Maximum unbound concentration in human plasma
*K*_m_: Michaelis-Menten constant
NAT: Nucleobase-ascorbate transporter
ORF: Open reading frame
OAT10: Organic anion transporter 10
SU-affecting drug: Serum urate-affecting drug
sgRNA: Single guide RNA
SVCT: Sodium-dependent vitamin C transporter
TKO: Triple knockout
URAT1: Urate transporter 1
UOX: Uricase
WT: Wild-type
