# Supplementary material for: Vitamin C transporter SVCT1 serves a physiological role as a urate importer: functional analyses and in vivo investigations
Source: Pflugers Arch. 2023 Feb 7;475(4):489–504. doi: 10.1007/s00424-023-02792-1 (PMC10011331; doi:10.1007/s00424-023-02792-1)
Supplement: Supplementary file 1 — Supplementary file1 (DOCX 272 KB) [file 424_2023_2792_MOESM1_ESM.docx]

***Pflügers Archiv - European Journal of Physiology***

**Supplementary Information**

**Vitamin C transporter SVCT1 serves a physiological role as a urate importer: functional analyses and *in vivo* investigations**

Yu Toyoda^1,†^, Hiroshi Miyata^1,†^, Naohiro Uchida^1,†^, Keito Morimoto^1^, Ryuichiro Shigesawa^1^, Hidetoshi Kassai^2^, Kazuki Nakao^2^, Naoko H Tomioka^3^, Hirotaka Matsuo^4^, Kimiyoshi Ichida^5^, Makoto Hosoyamada^3^, Atsu Aiba^2^, Hiroshi Suzuki^1^, Tappei Takada^1,*^

1. Department of Pharmacy, The University of Tokyo Hospital, 7-3-1 Hongo, Bunkyo-ku, Tokyo 113-8655, Japan
2. Laboratory of Animal Resources, Center for Disease Biology and Integrative Medicine, Graduate School of Medicine, The University of Tokyo, 7-3-1 Hongo, Bunkyo-ku, Tokyo 113-0033, Japan
3. Department of Human Physiology and Pathology, Faculty of Pharma-Sciences, Teikyo University, 2-11-1 Kaga, Itabashi-ku, Tokyo 173-8605, Japan
4. Department of Integrative Physiology and Bio-Nano Medicine, National Defense Medical College, 3-2 Namiki, Tokorozawa, Saitama 359-8513, Japan
5. Department of Pathophysiology, Tokyo University of Pharmacy and Life Sciences, 1432-1 Horinouchi, Hachioji, Tokyo 192-0392, Japan

^†^ Yu Toyoda, Hiroshi Miyata, and Naohiro Uchida contributed equally to this study.

^*^ **Correspondence to:**

Prof. Tappei Takada

Department of Pharmacy, The University of Tokyo Hospital, Tokyo, Japan; tappei-tky@umin.ac.jp

**Supplementary Figures S1–S5**

**Supplementary Tables S1–S5**

**Supplementary References**

**Supplementary Figures**

**Supplementary Fig. S1. Confirmation of *Svct1* knockout at the protein level.** Immunoblotting using an anti-Svct1 antibody revealed the renal expression of Svct1 in wild-type (WT) mice but not in *Svct1* knockout (KO) mice. Na^+^/K^+^ ATPase, a marker for plasma membrane components.

**Supplementary Fig. S2. Apical localization of human SVCT1 and mouse Svct1 expressed in polarized MDCKII cells.** Representative confocal microscopic Z-sectioning images of SVCT1 and Svct1 fused with EGFP are shown. Nuclei were stained using TO-PRO-3 iodide (gray). Bars: 10 μm.

**Supplementary Fig. S3. Effects of serum urate-affecting drugs and their active metabolites on the urate and vitamin C transport activities of SVCT1.** Forty-eight h after transfection, SVCT1-expressing 293A cells were subjected to a cell-based urate or vitamin C (VC) transport assay. The transport activities were measured for 2.5 min in the presence of each compound at experimentally maximum concentrations (refer to **Supplementary Table S2**). Values are shown as % of vehicle control (0.1% dimethyl sulfoxide); data are expressed as the mean ± SEM; *n* = 4. Data regarding the effects of each compound on SVCT1-mediated urate transport activities, are from **Fig. 6a**.

**Supplementary Fig. S4. Gene-expression patterns of renal urate re-absorbers in microdissected mouse renal tubule segments.** Data are obtained from a previous study [1]. Svct1, sodium-dependent vitamin C transporter 1; Urat1, urate transporter 1; Oat10, organic anion transporter 10; Hprt, hypoxanthine-guanine phosphoribosyl transferase (a housekeeping gene). PTS1, the initial segment of the proximal tubule; PTS2, proximal straight tubule in cortical medullary rays; PTS3, last segment of the proximal straight tubule in the outer stripe of outer medulla; DTL1, the short descending limb of the loop of Henle; DTL2, long descending limb of the loop of Henle in the outer medulla; DTL3, long descending limb of the loop of Henle in the inner medulla; ATL, ascending thin limb of Henle; MTAL, medullary ascending limb of Henle; CTAL, cortical ascending thin limb of Henle; DCT, distal convoluted tubule; CNT, connecting tubule; CCD, cortical collecting duct; OMCD, outer medullary collecting duct; IMCD, inner medullary collecting duct.

**Supplementary Fig. S5. Effects of acidic pH on SVCT1-mediated vitamin C and urate transport activities.** All uptake assays were conducted using transiently SVCT1-expressing 293A cells 48 h after plasmid transfection in Krebs–Ringer buffer. Experimental conditions: pH 7.4 or pH 6.4; incubation time, 2.5 min; [1-^14^C]-vitamin C (VC) in the transport buffer, 20 μM; [8-^14^C]-urate in the transport buffer, 10 μM. Values are shown as % of control (pH 7.4 for each SVCT1 substrate). Data are expressed as the mean ± SD; *n* = 3–4. ^††^, *P* < 0.01 (two-sided *t*-test).

**Supplementary Tables**

**Supplementary Table S1. Key Resources.**

| **REAGENT or RESOURCE** | **SOURCE** | **IDENTIFIER** |
| --- | --- | --- |
| ***Antibodies*** | | |
| Rabbit polyclonal anti-EGFP | Life Technologies | Cat# A11122; RRID: AB_221569; 1:1,000 dilution |
| SVCT1 (D-19) (goat polyclonal anti-mouse Svct1) | Santa Cruz Biotechnology | Cat# sc-9921; RRID: AB_2302143; 1:100 dilution |
| Rabbit polyclonal anti-Na^+^/K^+^-ATPase α antibody | Santa Cruz Biotechnology | Cat# sc-28800; RRID: AB_2290063; 1:1,000 dilution |
| Donkey anti-rabbit IgG-horseradish peroxidase (HRP)-conjugate | GE Healthcare | Cat# NA934V; RRID: AB_772206; 1:2,500 dilution |
| Donkey anti-Goat IgG HRP affinity purified PAb antibody | R&D Systems | Cat# HAF109; RRID: AB_357236; 1:1,000 dilution |
| ***Chemicals*** | | |
| [8-^14^C]-Uric acid (53 mCi/mmol) | American Radiolabeled Chemicals | Cat# ARC0513 |
| Uric acid | FUJIFILM Wako Pure Chemical | Cat# 210-00225; CAS: 69-93-2 |
| Ascorbic acid, L-[1-^14^C]-(Vitamin C) | PerkinElmer | Cat# NEC146 |
| L(+)-Ascorbic acid | FUJIFILM Wako Pure Chemical | Cat# 012-04802; CAS: 50-81-7 |
| Acyclovir | FUJIFILM Wako Pure Chemical | Cat# 019-17421; CAS: 59277-89-3 |
| Polyethelenimine “MAX” (PEI-MAX) | Polysciences | Cat# 24765; CAS: 49553-93-7 |
| Clear-sol II | Nacalai Tesque | Cat# 09136-83 |
| Dimethyl sulfoxide | Nacalai Tesque | Cat# 13445-74; CAS: 67-68-5 |
| 6-Hydroxy benzbromarone | Alsachim | Cat# C7859; CAS: 152831-00-0 |
| Allopurinol | FUJIFILM Wako Pure Chemical | Cat# 019-12502; CAS: 315-30-0 |
| Atorvastatin calcium salt trihydrate | Tokyo Chemical Industry | Cat# A2476; CAS: 344423-98-9 |
| Benzbromarone | FUJIFILM Wako Pure Chemical | Cat# 028-15851; CAS: 3562-84-3 |
| Chlorothiazide | Tokyo Chemical Industry | Cat# C2259; CAS: 58-94-6 |
| Cyclosporin A | FUJIFILM Wako Pure Chemical | Cat# 035-16303; CAS: 59865-13-3 |
| Dotinurad | Kindly provided by Fuji Yakuhin | FYU-981; CAS: 1285572-51-1 |
| Ethambutol dihydrochloride | LKT Labs | Cat# E7230; CAS: 1070-11-7 |
| Febuxostat | Tokyo Chemical Industry | Cat# F0847; CAS: 144060-53-7 |
| Fenofibrate | Merck | Cat# F6020; CAS: 49562-28-9 |
| D(−)-Fructose | FUJIFILM Wako Pure Chemical | Cat# 123-02762; CAS: 57-48-7 |
| Furosemide | FUJIFILM Wako Pure Chemical | Cat# 068-01881; CAS: 54-31-9 |
| Hydrochlorothiazide | FUJIFILM Wako Pure Chemical | Cat# 080-06262; CAS: 58-93-5 |
| Lesinurad | Merck | Cat# SML1607; CAS: 878672-00-5 |
| Losartan potassium | LKT Labs | Cat# L5873; CAS: 124750-99-8 |
| Mizoribine | Tokyo Chemical Industry | Cat# M2399; CAS: 50924-49-7 |
| Nicotinic acid | FUJIFILM Wako Pure Chemical | Cat# 142-01232; CAS: 59-67-6 |
| Oxipurinol | FUJIFILM Wako Pure Chemical | Cat# 151-02761; CAS: 2465-59-0 |
| Probenecid | Merck | Cat# P8761; CAS: 57-66-9 |
| Pyrazinamide | Fisher Scientific Acros | Cat# AC157640250; CAS: 98-96-4 |
| Pyrazinecarboxylic acid | Tokyo Chemical Industry | Cat# P0940; CAS: 98-97-5 |
| Ribavirin | Tokyo Chemical Industry | Cat# R0077; CAS: 36791-04-5 |
| Rosuvastatin calcium salt | FUJIFILM Wako Pure Chemical | Cat# 187-03361; CAS: 147098-20-2 |
| Salicylic acid | FUJIFILM Wako Pure Chemical | Cat# 194-14862; CAS: 69-72-7 |
| Tacrolimus monohydrate | Tokyo Chemical Industry | Cat# M2258; CAS: 109581-93-3 |
| Theophylline | FUJIFILM Wako Pure Chemical | Cat# 209-09932; CAS: 58-55-9 |
| Topiroxostat | MedChemExpress | Cat# HY-14874; CAS: 577778-58-6 |
| Xylitol | Tokyo Chemical Industry | Cat# X0018; CAS: 87-99-0 |
| ***Critical Commercial Assays*** | | |
| Pierce^TM^ BCA Protein Assay Reagent A & B | Thermo Fisher Scientific | Cat# 23223, Cat# 23224 |

**Supplementary Table S1 (*continued*).**

| ***Recombinant DNA*** | | |
| --- | --- | --- |
| The complete human SVCT1 cDNA | This paper | NCBI Ref Sequence: NM_005847.5 |
| The complete mouse Svct1 cDNA | This paper | NCBI Ref Sequence: NM_011397.4 |
| The complete human URAT1 cDNA | Toyoda et al., (2020) [2] | NCBI Ref Sequence: NM_144585.3 |
| The complete human GLUT9a cDNA | Toyoda et al., (2016) [3] | NCBI Ref Sequence: NM_020041.2 |
| pCAG-EGxxFP | Mashiko et al., (2013) [4] | http://n2t.net/addgene:50716 ; RRID:Addgene_50716 |
| pCAG-EGxxFP-Cetn1 | Mashiko et al., (2013) [4] | http://n2t.net/addgene:50717 ; RRID:Addgene_50717 |
| pX330-Cetn1/1 | Mashiko et al., (2013) [4] | http://n2t.net/addgene:50718 ; RRID:Addgene_50718 |
| ***Virus strains*** | | |
| EGFP-expressing adenovirus | Toyoda et al., (2016) [3] | N/A |
| EGFP-ABCG2-expressing adenovirus | Ito et al., (2015) [5] | N/A |
| ***Experimental Models: Cell Lines*** | | |
| Human: HEK293 cells | Toyoda et al., (2016) [3] | N/A |
| Human: 293A cells | Invitrogen | R70507 |
| Dog: MDCKII cells | Toyoda et al., (2016) [3] | N/A |
| ***Experimental Models: Organisms/Strains*** | | |
| Mouse: C57BL/6J | Japan SLC | C57BL/6JJmsSlc |
| Mouse: B6;129S7-Uox^tm1Bay^/J | The Jackson Laboratory | JAX: 0022238 |
| Mouse: Svct1^19del^ (*Svct1* knockout) | This study | N/A |
| Mouse: *Urat1*-*Uox* double knockout | Hosoyamada et al., (2016) [6] | N/A |
| Mouse: *Svct1*-*Urat1*-*Uox* triple knockout | This study | N/A |
| ***Oligonucleotides*** | | |
| A full list of primers | This paper | See **Supplementary Table S3** |
| sgRNA for knockout of *Svct1* gene | This paper | See ***Materials and Methods*** |
| ***Deposited data*** | | |
| Mouse renal tubule gene expression | Chen et al., (2021) [1] | https://doi.org/10.1681/ASN.2020101406 |
| ***Software and Algorithms*** | | |
| Excel 2019 | Microsoft | https://products.office.com/ja-jp/home |
| Statcel4 add-in software | OMS Publishing | http://www.oms-publ.co.jp/ |
| GraphPad Prism 8 | GraphPad Software | https://www.graphpad.com/ |
| MassLynx NT software version 4.1 | Waters | https://www.waters.com/nextgen/jp/ja.html |

**Supplementary Table S2. List of serum urate-affecting drugs and their active metabolites used in this study.**

| **Effect on serum urate levels** | **Drugs** | **[μM]^*^** | **References** |
| --- | --- | --- | --- |
| *Decrease expectedly* | *Urate synthesis inhibitors* |  |  |
|  | Allopurinol | 300 | Watts *et al*., 1965 [7] |
|  | Oxypurinol | 100 | Watts *et al*., 1965 [7] |
|  | Febuxostat | 10 | Osada *et al*., 1993 [8] |
|  | Topiroxostat | 3 | Okamoto *et al*., 2003 [9] |
|  | *Uricosuric agents* |  |  |
|  | Benzbromarone | 100 | Enomoto *et al*., 2002 [10] |
|  | 6-Hydroxybenzbromarone | 100 | Oikawa *et al.*, 2005 [11] |
|  | Dotinurad | 100 | Taniguchi *et al*., 2019 [12] |
|  | Lesinurad | 30 | Miner *et al*., 2016 [13] |
|  | Probenecid | 100 | Gutman *et al*., 1954 [14] |
| *Decrease unexpectedly* | Atorvastatin | 30 | Ogata *et al.*, 2010 [15] |
|  | Fenofibrate | 300 | Harvengt *et al*., 1980 [16] |
|  | Losartan | 300 | Kamper and Nielsen, 2001 [17] |
|  | Rosuvastatin | 100 | Ogata *et al.*, 2010 [15] |
| *Increase unexpectedly* | Chlorothiazide | 300 | Kelley *et al.*, 1973 [18] |
|  | Cyclosporine | 30 | Palestine *et al.*, 1984 [19] |
|  | Ethambutol | 1000 | Postlethwaite *et al.*, 1972 [20] |
|  | Fructose | 1000 | Thomas *et al.*, 1975 [21] |
|  | Furosemide | 300 | Kelley *et al.*, 1973 [18] |
|  | Hydrochlorothiazide | 1000 | Healey *et al.*, 1959 [22] |
|  | Mizoribine | 300 | Ishikawa *et al.*, 1986 [23] |
|  | Nicotinic acid | 300 | Cruess-Callaghan and FitzGerald, 1966 [24] |
|  | Pyrazinamide | 300 | Cullen *et al.*, 1956 [25] |
|  | Pyrazinecarboxylic acid | 1000 | Cullen *et al.*, 1956 [25] |
|  | Ribavirin | 1000 | Yamashita *et al.*, 2008 [26] |
|  | Salicylic acid | 1000 | Yu and Gutman, 1959 [27] |
|  | Tacrolimus | 30 | Kanbay *et al.*, 2005 [28] |
|  | Theophylline | 100 | Yamamoto *et al.*, 1991 [29] |
|  | Xylitol | 1000 | Yamamoto *et al.*, 1991 [29] |

^*^ Maximum concentrations used in this study.

This is the updated version of a previous paper [30], incorporating new information.

**Supplementary Table S3. Primer sequences for genotyping of each knockout allele.**

| **Primers** | **Nucleotide sequences (5′ to 3′)** | **Amplicon (bp)** |
| --- | --- | --- |
| *Svct1* KO |  |  |
| Forward (for WT) | ggacaccatgattgcaccct | 213 |
| Forward (for KO) | actggacaccatgattgcaa | 194 |
| Reverse (common) | ggagtatgcgtctcaacacc |  |
| *Urat1* KO |  |  |
| Forward (common) | gacttctctctccaccctcctt |  |
| Reverse (for WT) | ccatgggtttctcctgggtacc | 313 |
| Reverse (for KO) | gggtgttgggtcgtttgttcgg | 225 |
| *Uox* KO |  |  |
| Forward (for WT) | tcgagacctttgcaatgaacatc | 276 |
| Forward (for KO) | cgccttctatcgccttcttgacg | 150 |
| Reverse (common) | ttctcatctgctccacctcacag |  |

WT, wild-type; KO, knockout.

For the genotyping of each KO allele, all (three) primers were used in one reaction tube.

Thermal cycling conditions for genotyping: 95°C for 2 min; 28 cycles of 95°C for 30 s, 62°C for 10 s, 72°C for 15 s; 5 min at 72°C (for *Svct1* KO; representative band patterns of each amplicon are shown in **Fig. 4c**): 95°C for 2 min; 30 cycles of 95°C for 30 s, 62°C for 30 s, 72°C for 20 s; 5 min at 72°C (for *Urat1* KO): 95°C for 2 min; 30 cycles of 95°C for 30 s, 60°C for 30 s, 72°C for 20 s; 5 min at 72°C (for *Uox* KO).

**Supplementary Table S4. Monitoring parameters in LC-MS/MS analyses.**

| **Compounds** | **Retention time [min]** | **Ionization mode** | **Monitor ion [*m*/*z*]** | | **Cone [V]** | **Collision [eV]** |
| --- | --- | --- | --- | --- | --- | --- |
|  |  |  | **Precursor** | **Product** |  |  |
| Urate | 1.39 | ESI negative | 167.00 | 124.00 | 38 | 13 |
| Acyclovir for urate | 3.35 | ESI negative | 224.20 | 150.10 | 40 | 17 |
| Creatinine | 1.45 | ESI positive | 113.90 | 43.94 | 30 | 30 |
| Acyclovir for creatinine | 2.00 | ESI positive | 226.07 | 135.04 | 14 | 30 |

**Supplementary Table S5**. **Plasma concentration-based interaction scores to estimate the potential inhibition of urate or vitamin C transport of SVCT1 by each tested compound.** As an interaction score, we determined the value of f_u_C_max_/IC_50_; a high score reflects the possibility of inhibition by tested compounds at clinical doses in humans.

| **Compounds** | **f_u_ ^*^** | **C_max_ [μM] ^*^** | **f_u_C_max_ [μM]** | **with urate** | |  | **with vitamin C** | |
| --- | --- | --- | --- | --- | --- | --- | --- | --- |
|  |  |  |  | **IC_50_ [μM]** | **Interaction scores [f_u_C_max_/IC_50_]** |  | **IC_50_ [μM]** | **Interaction scores [f_u_C_max_/IC_50_]** |
| Benzbromarone | 0.037 | 5.4 | 2.0 × 10^−1^ | 9.8 | 2.04 × 10^−2^ |  | 8.4 | 2.24 × 10^−2^ |
| 6-Hydroxybenzbromarone | N/A | N/A | N/A | 10.0 | N/A |  | 2.9 | N/A |
| Dotinurad | 0.007 | 1.2 | 8.4 × 10^−3^ | 59.6 | 1.41 × 10^−4^ |  | 11.8 | 7.12 × 10^−4^ |

^*^ These values are obtained from previous studies [12, 30]. IC_50_, the half-maximal inhibitory concentration values of target compounds against urate or vitamin C transport by SVCT1; f_u_, the fraction of drug unbound in human plasma; C_max_, maximum concentration in human plasma; f_u_C_max_, maximum unbound concentration in human plasma; N/A, not available.

**Supplementary References**

1. Chen L, Chou CL, and Knepper MA (2021) A Comprehensive Map of mRNAs and Their Isoforms across All 14 Renal Tubule Segments of Mouse. J Am Soc Nephrol 32(4):897-912. <https://doi.org/10.1681/ASN.2020101406>

2. Toyoda Y, Takada T, Saito H, Hirata H, Ota-Kontani A, Kobayashi N, Tsuchiya Y, and Suzuki H (2020) Inhibitory effect of Citrus flavonoids on the in vitro transport activity of human urate transporter 1 (URAT1/SLC22A12), a renal re-absorber of urate. NPJ Sci Food 4:3. <https://doi.org/10.1038/s41538-020-0063-7>

3. Toyoda Y, Takada T, Miyata H, Ishikawa T, and Suzuki H (2016) Regulation of the Axillary Osmidrosis-Associated ABCC11 Protein Stability by N-Linked Glycosylation: Effect of Glucose Condition. PLoS One 11(6):e0157172. <https://doi.org/10.1371/journal.pone.0157172>

4. Mashiko D, Fujihara Y, Satouh Y, Miyata H, Isotani A, and Ikawa M (2013) Generation of mutant mice by pronuclear injection of circular plasmid expressing Cas9 and single guided RNA. Sci Rep 3:3355. <https://doi.org/10.1038/srep03355>

5. Ito N, Ito K, Ikebuchi Y, Toyoda Y, Takada T, Hisaka A, Oka A, and Suzuki H (2015) Prediction of Drug Transfer into Milk Considering Breast Cancer Resistance Protein (BCRP)-Mediated Transport. Pharm Res 32(8):2527-2537. <https://doi.org/10.1007/s11095-015-1641-2>

6. Hosoyamada M, Tsurumi Y, Hirano H, Tomioka NH, Sekine Y, Morisaki T, and Uchida S (2016) Urat1-Uox double knockout mice are experimental animal models of renal hypouricemia and exercise-induced acute kidney injury. Nucleosides Nucleotides Nucleic Acids 35(10-12):543-549. <https://doi.org/10.1080/15257770.2016.1143559>

7. Watts RW, Watts JE, and Seegmiller JE (1965) Xanthine oxidase activity in human tissues and its inhibition by allopurinol (4-hydroxypyrazolo[3,4-d] pyrimidine). J Lab Clin Med 66(4):688-697. <https://doi.org/10.5555/uri:pii:0022214365900533>

8. Osada Y, Tsuchimoto M, Fukushima H, Takahashi K, Kondo S, Hasegawa M, and Komoriya K (1993) Hypouricemic effect of the novel xanthine oxidase inhibitor, TEI-6720, in rodents. Eur J Pharmacol 241(2-3):183-188. <https://doi.org/10.1016/0014-2999(93)90201-r>

9. Okamoto K, Eger BT, Nishino T, Kondo S, Pai EF, and Nishino T (2003) An extremely potent inhibitor of xanthine oxidoreductase. Crystal structure of the enzyme-inhibitor complex and mechanism of inhibition. J Biol Chem 278(3):1848-1855. <https://doi.org/10.1074/jbc.M208307200>

10. Enomoto A, Kimura H, Chairoungdua A, et al (2002) Molecular identification of a renal urate anion exchanger that regulates blood urate levels. Nature 417(6887):447-452. <https://doi.org/10.1038/nature742>

11. Oikawa T, Kunishima C, Matsumoto K, Ishikawa S, and Endou H (2005) The inhibitory effect of benzbromarone and 6-hydroxybenzbromarone on urate transporter (URAT1). J New Rem & Clin 54(6):15-20.

12. Taniguchi T, Ashizawa N, Matsumoto K, et al (2019) Pharmacological Evaluation of Dotinurad, a Selective Urate Reabsorption Inhibitor. J Pharmacol Exp Ther 371(1):162-170. <https://doi.org/10.1124/jpet.119.259341>

13. Miner JN, Tan PK, Hyndman D, et al (2016) Lesinurad, a novel, oral compound for gout, acts to decrease serum uric acid through inhibition of urate transporters in the kidney. Arthritis Res Ther 18(1):214. <https://doi.org/10.1186/s13075-016-1107-x>

14. Gutman AB, Yu TF, and Randolph V (1954) Further Observations on the Uricosuric Effects of Probenecid (Benemid) in Tophaceous Gout. Trans Assoc Am Physicians 67:250-260.

15. Ogata N, Fujimori S, Oka Y, and Kaneko K (2010) Effects of three strong statins (atorvastatin, pitavastatin, and rosuvastatin) on serum uric acid levels in dyslipidemic patients. Nucleosides Nucleotides Nucleic Acids 29(4-6):321-324. <https://doi.org/10.1080/15257771003741323>

16. Harvengt C, Heller F, and Desager JP (1980) Hypolipidemic and Hypouricemic Action of Fenofibrate in Various Types of Hyperlipoproteinemias. Artery 7(1):73-82.

17. Kamper A, and Nielsen AH (2001) Uricosuric effect of losartan in renal transplanted patients. Transplant Proc 33(1-2):1201. <https://doi.org/10.1016/s0041-1345(00)02385-x>

18. Kelley WN, Grobner W, and Holmes E (1973) Current concepts in the pathogenesis of hyperuricemia. Metabolism 22(7):939-959.

19. Palestine AG, Nussenblatt RB, and Chan CC (1984) Side effects of systemic cyclosporine in patients not undergoing transplantation. Am J Med 77(4):652-656.

20. Postlethwaite AE, Bartel AG, and Kelley WN (1972) Hyperuricemia due to ethambutol. N Engl J Med 286(14):761-762. <https://doi.org/10.1056/NEJM197204062861407>

21. Thomas DW, Edwards JB, and Edwards RG (1975) Side effects of sugar substitutes during intravenous administration. Nutr Metab 18 Suppl 1:227-241.

22. Healey LA, Magid GJ, and Decker JL (1959) Uric acid retention due to hydrochlorothiazide. N Engl J Med 261:1358-1362. <https://doi.org/10.1056/NEJM195912312612702>

23. Ishikawa I, Maekawa S, Saito T, Horiguchi T, Shinoda A, and Ishii H (1986) [Mizoribine-induced hyperuricemia]. Nihon Jinzo Gakkai shi 28(10):1353-1357.

24. Cruess-Callaghan A, and FitzGerald O (1966) A mechanism of nicotinic acid-induced hyperuricaemia. Ir J Med Sci 6(491):484-487.

25. Cullen JH, Early LJ, and Fiore JM (1956) The occurrence of hyperuricemia during pyrazinamide-isoniazid therapy. American review of tuberculosis 74(2 Part 1):289-292.

26. Yamashita N, Enjoji M, Kotoh K, et al (2008) Investigation of hyperuricemia during pegylated-interferon-alpha2b plus ribavirin combination therapy in patients with chronic hepatitis C. J Dig Dis 9(1):27-31. <https://doi.org/10.1111/j.1443-9573.2007.00316.x>

27. Yu TF, and Gutman AB (1959) Study of the paradoxical effects of salicylate in low, intermediate and high dosage on the renal mechanisms for excretion of urate in man. J Clin Invest 38(8):1298-1315. <https://doi.org/10.1172/JCI103905>

28. Kanbay M, Akcay A, Huddam B, Usluogullari CA, Arat Z, Ozdemir FN, and Haberal M (2005) Influence of cyclosporine and tacrolimus on serum uric acid levels in stable kidney transplant recipients. Transplant Proc 37(7):3119-3120. <https://doi.org/10.1016/j.transproceed.2005.08.042>

29. Yamamoto T, Moriwaki Y, Suda M, Takahashi S, Hiroishi K, and Higashino K (1991) Theophylline-induced increase in plasma uric acid--purine catabolism increased by theophylline. Int J Clin Pharmacol Ther Toxicol 29(7):257-261.

30. Miyata H, Takada T, Toyoda Y, Matsuo H, Ichida K, and Suzuki H (2016) Identification of Febuxostat as a New Strong ABCG2 Inhibitor: Potential Applications and Risks in Clinical Situations. Front Pharmacol 7:518. <https://doi.org/10.3389/fphar.2016.00518>
